# Supplementary material for: Yiai Fuzheng decoction inhibits triple-negative breast cancer by remodeling the immune microenvironment
Source: Front Immunol. 2025 Sep 30;16:1615631. doi: 10.3389/fimmu.2025.1615631 (PMC12518410; doi:10.3389/fimmu.2025.1615631)
Supplement: Supplementary file 2 [file DataSheet1.zip › Supplementary File 2/Treatment/C_vs_D/07_Pathway_Analysis/Pathway_Analysis/Sig_Pathways_Name/Galactose metabolism.pdf]

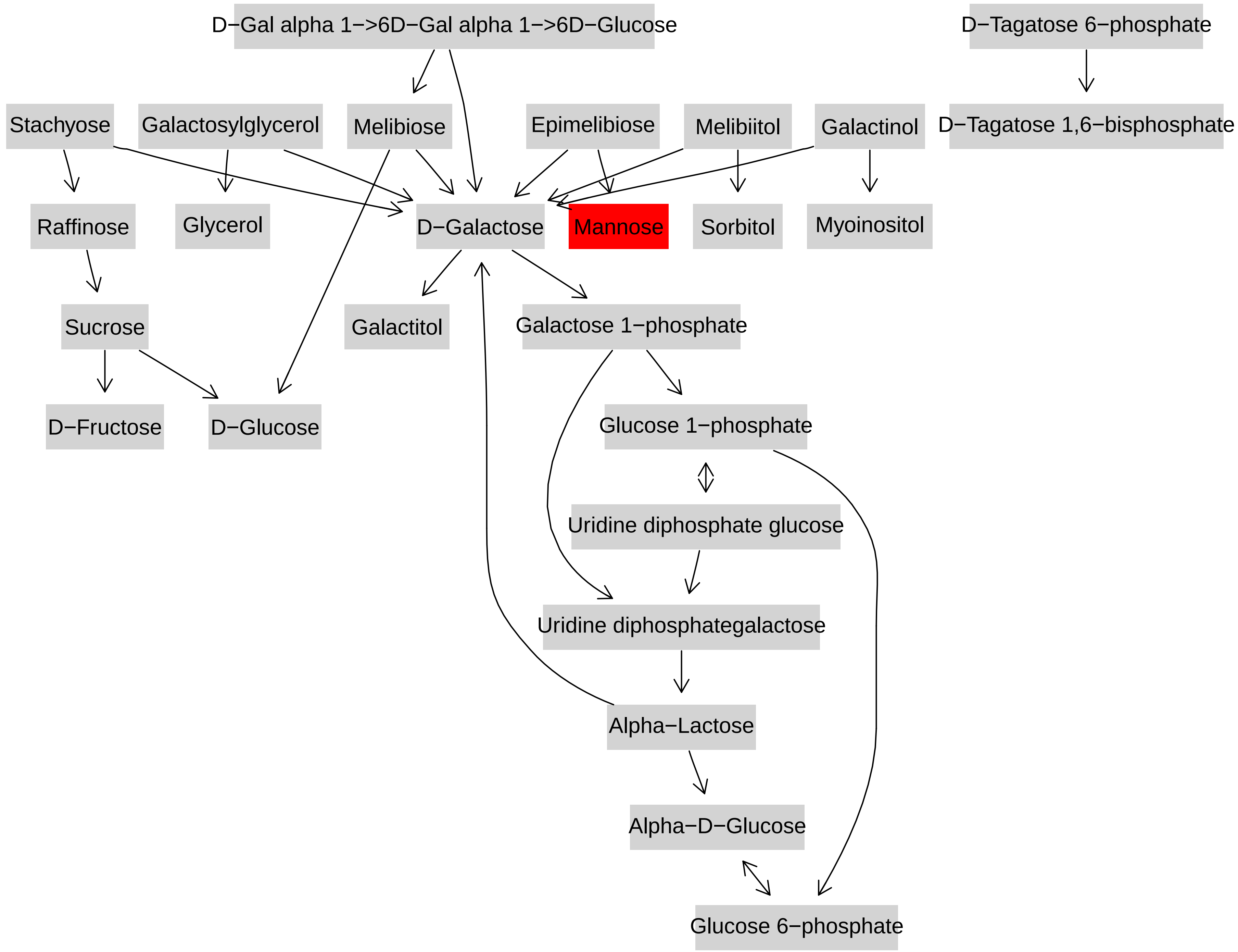

D−Gal alpha 1−>6D−Gal alpha 1−>6D−Glucose
D−Tagatose 6−phosphate
Stachyose
Galactosylglycerol
Melibiose
Epimelibiose
Melibiitol
Galactinol
D−Tagatose 1,6−bisphosphate
Raffinose
Glycerol
D−Galactose
Mannose
Sorbitol
Myoinositol
Sucrose
Galactitol
Galactose 1−phosphate
D−Fructose
D−Glucose
Glucose 1−phosphate
Uridine diphosphate glucose
Uridine diphosphategalactose
Alpha−Lactose
Alpha−D−Glucose
Glucose 6−phosphate
